# Fine Mapping and Candidate Genes Analysis for Regulatory Gene of Anthocyanin Synthesis in the Corolla, Shedding Light on Wild Potato Evolution

**DOI:** 10.3390/ijms26051966

**Published:** 2025-02-25

**Authors:** Zewei Zhang, Hongjun Li, Lingkui Zhang, Shaoguang Duan, Guangcun Li, Yanfeng Duan

**Affiliations:** State Key Laboratory of Vegetable Biobreeding, Key Laboratory of Biology and Genetic Improvement of Tuber and Root Crop of Ministry of Agriculture and Rural Affairs, Institute of Vegetables and Flowers, Chinese Academy of Agricultural Sciences, Beijing 100081, China; zzw19134128565@163.com (Z.Z.); hongjunlisc@163.com (H.L.); zhanglingkui960127@163.com (L.Z.); duanshaoguang@caas.cn (S.D.)

**Keywords:** corolla color, fine mapping, anthocyanin, regulatory, potato

## Abstract

*Petota* includes more than 100 species (wild and cultivated), presenting a rich variety of corolla colors and associated traits. This variability provides important opportunities for investigating the differentiation of orthologous genes’ functions and their evolutionary pathways. However, the genetic underpinnings of this diversity in corolla colors are still to be further explored. In our previous study, a locus responsible for corolla color in potato was mapped to a 740 kb region on chromosome 10, which contains the *AN2* gene previously identified as a regulation gene for corolla color. In the present study, this locus was further refined to a 380 kb interval through recombinant analysis. Targeted analysis of anthocyanidins and carotenoids revealed that purple corollas exhibit significantly higher levels of petunidin and delphinidin, while showing significantly lower levels of lutein and β-carotene compared to yellow corollas. Transcriptome and qRT-PCR analysis indicated that *StMYB180*, rather than *AN2*, is the candidate gene responsible for regulating coloration, specifically on the abaxial side of the corolla in potato. Expression analysis revealed that *StMYB180* is exclusively highly expressed in corolla and leaf tissues, with purple coloration on the abaxial side of both corollas and leaves. Phylogenetic analysis further suggests that corolla color-regulatory genes may be closely tied to the origin and evolutionary trajectory of potato species. This study provides valuable insights into the regulation of tissue-specific expression of anthocyanin biosynthesis in potato and lays the groundwork for understanding the evolution of orthologous genes in the *Petota* section.

## 1. Introduction

Potato (*Solanum tuberosum* L.) is a member of the *Petota* section of the *Solanum* genus, within the Solanaceae family, and is regarded as the world’s most important non-cereal food crop [1]. The *Petota* section includes 107 wild species and four cultivated species, representing a diverse and accessible germplasm resource that surpasses that of any other major crop [2,3]. Wild potato species exhibit ploidy levels ranging from diploid to hexaploid and reproduce in nature through sexual and clonal propagation.

**Figure 1:**
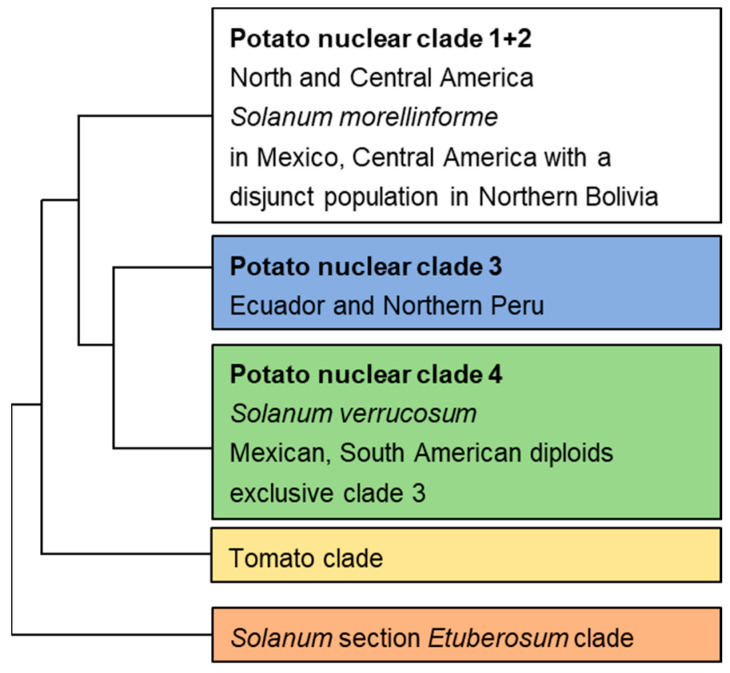
Phylogenetic analysis of the diploid species within *Solanum* section *Petota*, illustrating three nuclear clades alongside outgroups (tomato and *Etuberosum*) adapted from Spooner et al. [3]. In clades 1 and 2, diploid species are predominantly characterized by white corollas. In clade 3, diploid species exhibit blue to purple corollas. Clade 4 encompasses diploid species with a variety of corolla colors.

Corolla color is a key visual signal for insect-pollinated plants and plays a crucial role in plant reproduction [4]. Pollinator-mediated selection has long been considered a significant factor in the origin and maintenance of species diversity in plants [5]. Therefore, corolla color is an excellent model for studying the evolution and differentiation of angiosperms [6]. Molecular phylogenies classify the *Petota* section into three clades based on nuclear DNA data [3]. In clades 1 and 2, diploid species typically have white corollas, while clade 3 diploid species exhibit blue to purple corollas, and clade 4 diploid species display various corolla colors (Figure 1). Furthermore, corolla coloration is diverse; some species have uniformly colored corollas, while others exhibit predominant and secondary colors, with variations in the distribution of secondary colors. In some instances, coloration may only occur on the abaxial side of the corolla. This diverse coloration reflects high genetic variability, and the correlated clade-specific corolla colors suggest the presence of an evolutionary relationship among clades. However, the evolutionary relationship of this variation remains unclear, which hampers our ability to effectively protect and utilize potato resources.

The anthocyanin biosynthesis pathway is highly conserved and involves both structural enzymes and transcriptional regulators, which are often tissue specific. In cultivated diploid potato, three classic loci—*R*, *P*, and *I*—are known to be involved in anthocyanin production in tuber skin [7,8]. These loci have been genetically mapped to chromosomes 2, 11, and 10, respectively, and are likely allelic to the tetraploid *D*, *R*, and *P* loci [9]. Loci *R* and *P* are essential for producing red pelargonidin or blue/purple petunidin-based anthocyanin pigments throughout the potato plant, including tubers, flowers, sprouts, and stems. *R* encodes dihydroflavonol 4-reductase (DFR), while *P* encodes flavonoid 3′5′-hydroxylase (F3′5′H) [10,11]. The *P* locus is known to be epistatic to *R* [7]. The *I* (or *D*) locus is required for the tissue-specific expression of anthocyanins in tuber skin and encodes an R2R3-MYB transcription factor, Stan2, located on chromosome 10 [12]. Similarly, the *F* locus is necessary for anthocyanin synthesis in corolla and is tightly linked to the *I* locus [13,14]. The *F* locus encodes a MYB transcription factor named *StFlAN2,* which is a homolog of the *ANTHOCYANIN 2* (*AN2*) gene family [15]. The transfer of *StFlAN2* into white-flowered homozygous doubled-monoploid (DM) plants resulted in the recovery of uniformly colored purple corollas [15].

Previously, we mapped a locus responsible for anthocyanin synthesis in corollas in a wild potato F_1_ population to a 740 kb region on chromosome 10, which harbors the *AN2* gene [16]. The female parent (PH1-2) and half of the F_1_ progeny exhibit purple coloring on the abaxial side of the corollas, while the male parent (PH1-14) and the other half have uniformly yellow-colored corollas. In the present study, we narrowed the locus to a 380 kb interval using recombinant analysis. According to the annotation of the reference genome DMv8.1 [17], the locus contains 28 genes, including five MYB transcription factor genes: *DM8C10G21120*, *DM8C10G21130* (*AN2*), *DM8C10G21170*, *DM8C10G21180*, and *DM8C10G21200*, designated *StMYB120*, *StMYB130*, *StMYB170*, *StMYB180*, and *StMYB200*. There are no structural genes involved in the anthocyanin biosynthesis pathway located within the candidate locus. Based on the corolla phenotype, combined with metabolite determination and expression analysis, we predict that *StMYB180*, a *MYB113-like* gene referred to as *ScFL1*, is the candidate gene. This locus is involved in regulating the tissue-specific expression of anthocyanin biosynthesis on the abaxial side of the corollas in potato and is distinct from the *F* locus, hereafter termed *Fab*. This research provides new insights into the regulation of anthocyanin biosynthesis in potato and establishes a foundation for studying the evolution of orthologous genes in *Petota*.

## 2. Results

### 2.1. Anthocyanin and Carotenoid Accumulation Contribute to the Color Diversity of Potato Corollas

**Figure 2:**
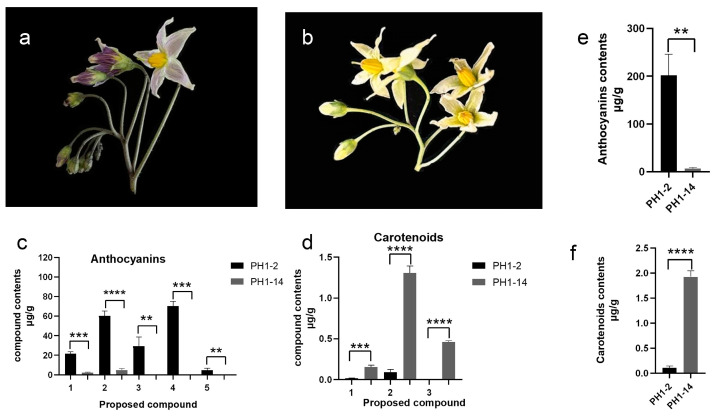
Analysis of corolla color phenotypes and associated anthocyanidins and carotenoids in PH1-2 and PH1-14. (**a**) PH1-2 exhibited purple coloration on the reverse side of the corollas. (**b**) PH1-14 displayed a uniform yellow coloration throughout the corollas. (**c**) Measurements of anthocyanidins in the corollas of PH1-2 and PH1-14: 1. delphinidin-3-(cis-p-coumaroyl)-rutinoside-5-glucoside; 2. delphinidin-3-(trans-p-coumaroyl)-rutinoside-5-glucoside; 3. petunidin-3-(cis-p-coumaroyl)-rutinoside-5-glucoside; 4. petunidin-3-(trans-p-coumaroyl)-rutinoside-5-glucoside; 5. petunidin-3-(feruloyl)-rutinoside-5-glucoside. (**d**) Measurement of carotenoids in the corollas of PH1-2 and PH1-14: 1. lutein; 2. β-carotene; 3. unknown component. (**e**) Quantification of total anthocyanins in the corollas of PH1-2 and PH1-14. (**f**) Quantification of total carotenoids in the corollas of PH1-2 and PH1-14. Error bars denote the SD from three biological replicates. Asterisks represent the statistical significance determined using Student’s *t*-test, where **** indicates *p* < 0.0001, *** indicates *p* < 0.001, and ** indicates *p* < 0.01.

Anthocyanins are water-soluble natural pigments widely distributed across various plant taxa, capable of producing a spectrum of colors ranging from orange/red to violet/blue. Carotenoids, predominantly C_40_ isoprenoid compounds, impart colors that range from yellow to red in fruits, flowers, and other plant organs. Two wild potato species genotypes, PH1-2 and PH1-14, were utilized as parents to construct an F_1_ population for mapping the *Fab* locus [16]. The purple coloration of the PH1-2 corollas is observed on the abaxial side of the corollas (Figure 2a), while the corollas of PH1-14 exhibit a uniform yellow coloration (Figure 2b). To identify specific types of anthocyanins and carotenoids contributing to flower coloration, we performed targeted measurements of these compounds in the corollas of PH1-2 and PH1-14.

In total, five types of anthocyanins, including two categories—petunidin and delphinidin glucosides—were detected in the corollas of PH1-2. All identified anthocyanins were significantly different compared to those in PH1-14 and are associated with the formation of purple corollas (Figure 2c). Delphinidin glucosides were present in both PH1-2 and PH1-14, while petunidin glucosides were exclusive to PH1-2. Notably, delphinidin-3-(trans-p-coumaroyl)-rutinoside-5-glucoside and petunidin-3-(trans-p-coumaroyl)-rutinoside-5-glucoside exhibited the highest levels in PH1-2 and are likely the primary contributors to the purple corolla coloration.

We identified three types of carotenoids: lutein, β-carotene, and an unidentified component closely resembling lycopene, all contributing to the formation of yellow corollas (Figure 2d). The unidentified component was detected only in PH1-14. Furthermore, all carotenoid components in PH1-14 were significantly higher than those in PH1-2, with β-carotene displaying the highest content and being the main pigment responsible for the yellow coloration of PH1-14 corollas. Substantial differences in the types and contents of anthocyanins and carotenoids between PH1-2 and PH1-14 correspond with their respective corolla phenotypes (Figure 2e,f).

### 2.2. ScFL1 Was Fine-Mapped in a 380 kb Interval on Chromosome 10

Previously, we mapped *ScFL1* to the distal end of chromosome 10, located between InDel538 (53.11 Mb) and InDel652 (53.85 Mb), using an F_1_ population generated from a cross between PH1-2 and PH1-14 [16]. To perform fine mapping of this gene, we developed a larger F_1_ segregation population consisting of 279 individuals from the same cross. We genotyped all 279 individuals using a set of 12 markers developed with the primer design module of OcBSA and identified 14 recombinants.

**Figure 3:**
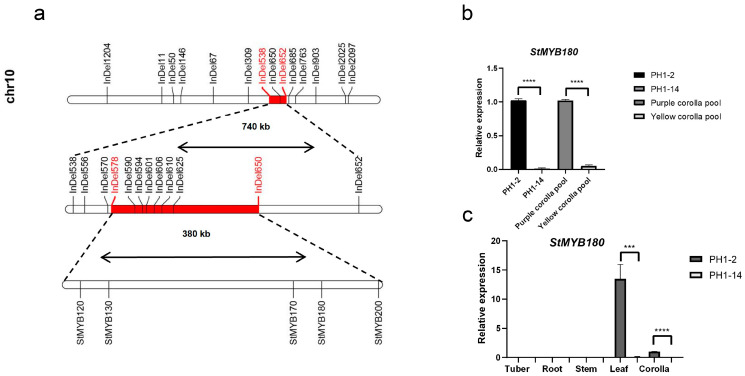
Fine mapping and expression analysis of *ScFL1.* (**a**) In the F_1_ population derived from the cross between PH1-2 and PH1-14, *ScFL1* was initially mapped to an interval between the markers InDel538 and InDel652, spanning 740 kb on the DMv8.1 reference genome (first bar from the top). Utilizing a larger F_1_ population from the same cross, the mapping interval for *ScFL1* was refined to approximately 380 kb with the flanking markers of the InDel578 at 53,214,383 bp and InDel650 at 53,594,067 bp on chromosome 10, according to the DMv8.1 reference genome (second bar from the top). The layout of MYB genes within the *Fab* locus is depicted in the bar at the bottom. (**b**) Relative expression of *StMYB180* in PH1-2, PH1-14, the purple and yellow corolla pools. (**c**) Relative expression of *StMYB180* across different tissues of PH1-2 and PH1-14. The *ef1α* gene was used as an internal control. Error bars denote the SD from three biological replicates. Asterisks represent the statistical significance determined using Student’s *t*-test, where **** indicates *p* < 0.0001, *** indicates *p* < 0.001.

Based on the analysis of four key recombinants, we localized *ScFL1* to a 380 kb interval between InDel578 (53.21 Mb) and InDel650 (53.59 Mb) according to the DMv8.1 reference genome [17] (Figure 3a, Table S1). Within this region, we identified 28 genes, including five MYB transcription factor genes: *StMYB120*, *StMYB130* (*AN2*), *StMYB170*, *StMYB180*, and *StMYB200*. Notably, no structural genes involved in anthocyanin biosynthesis were identified in this region (Figure 3a, Table S2).

### 2.3. StMYB180 Is Considered the Candidate Gene of ScFL1

To identify the candidate gene within the *Fab* locus, we pooled corolla tissues from purple and yellow individuals of the F_1_ population for Bulk Segregant RNA Sequencing (BSR-Seq), and generated genome-wide gene expression data, revealing a total of 3506 differentially expressed genes (DEGs), which comprises 1947 upregulated genes and 1559 downregulated genes. Notably, nine DEGs were located within the *Fab* locus, with three genes downregulated and six genes upregulated in purple corollas compared to yellow corollas (Table 1).

The relationship between corolla color and MYB transcription factors has been extensively documented in numerous flowering plants [18]. Within diploid cultivated potato, a homolog of the *ANTHOCYANIN 2* (*AN2*) gene family, referred to as *StFlAN2*, has been identified as a regulator of anthocyanin biosynthesis in corollas [15]. Among the five MYB genes located within the *Fab* locus—*StMYB120*, *StMYB130* (*AN2*), *StMYB170*, *StMYB180*, and *StMYB200*, *StMYB130* does not exhibit differential expression between the contrasting corolla color pools. In contrast, *StMYB120*, *StMYB170*, and *StMYB200* display significant differential expression, showing higher levels in the yellow corolla pool compared to the purple corolla pool. Given the established positive correlation between MYB gene expression and purple corolla pigmentation, these three genes are unlikely to be the primary candidates within the *Fab* locus. Conversely, *StMYB180* demonstrates elevated expression levels in purple corollas relative to yellow corollas, and quantitative PCR analysis further corroborates the expression of *StMYB180* in both parent plants and the two contrasting corolla pools (Figure 3b). These results suggest that *StMYB180* should be the candidate gene for the *Fab* locus, leading to its designation as *ScFL1*.

To investigate the expression pattern of *StMYB180*, qRT-PCR analysis was conducted on tissues from PH1-2 and PH1-14, including tubers, roots, stems, leaves, and corollas. While no visible differences in tuber skin or root coloration were observed between PH1-2 and PH1-14, distinct variations were evident in other tissues. PH1-2 exhibited green stems and purple abaxial leaf coloration, whereas PH1-14 had purple stems and uniformly green leaves (Figure S1). The qRT-PCR results demonstrated significantly higher expression levels of *StMYB180* in the leaves and corollas of PH1-2 compared to PH1-14. In contrast, *StMYB180* expression was either absent or at minimal levels in tubers, roots, and stems of both genotypes (Figure 3c). These findings strongly suggest that *StMYB180* is a candidate regulatory gene involved in tissue-specific anthocyanin biosynthesis in the abaxial regions of corollas and leaves in potato.

### 2.4. Phylogenetic Analysis Suggests That Corolla Color-Regulating Gene(s) Are Associated with the Origin and Evolution of Wild and Cultivated Potatoes

**Figure 4:**
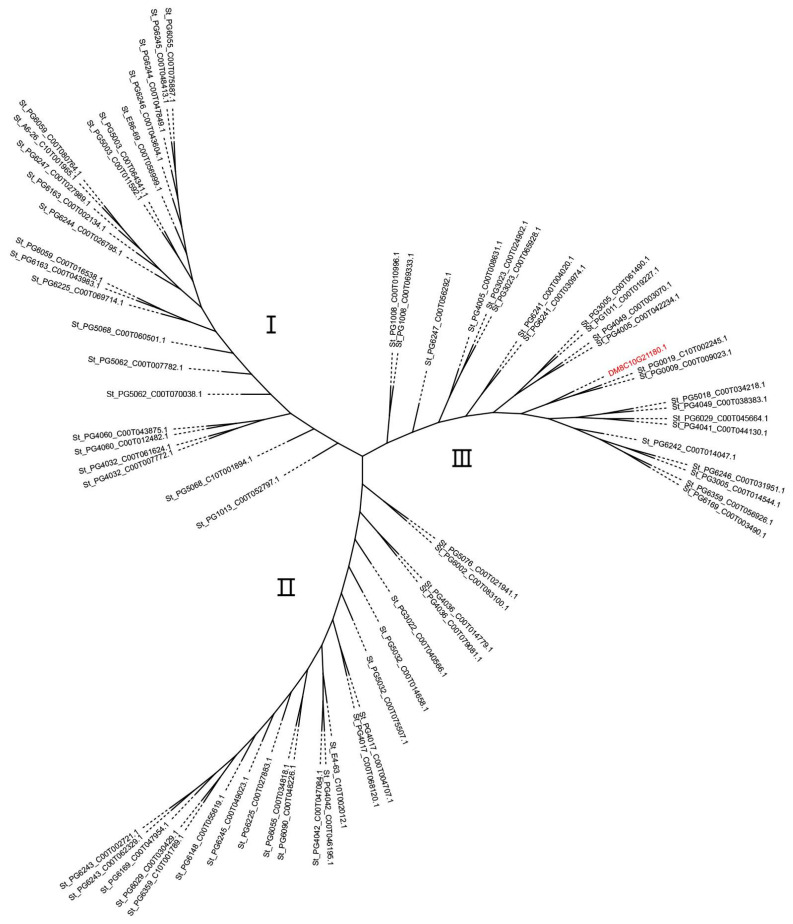
Phylogenetic analysis of *StMYB180* homologs. Seventy orthologs of *StMYB180* across 44 accessions, encompassing 23 wild, 19 cultivated, and 2 *Etuberosum* accessions were performed phylogenetic analysis and classified into three branches: I, II, and III with *DM8C10G21180.1* (*StMYB180*) marked in red.

To further explore the evolutionary implications of corolla color regulation, phylogenetic analysis was performed on *StMYB180* orthologs from 44 genome-assembled accessions [19], including 23 wild species, 19 landraces, and two species from the neighboring *Etuberosum* section (Table S4). A total of 70 orthologous genes were analyzed and classified into three branches: I, II, and III (Figure 4). Notably, landraces were distributed across all three branches, suggesting possible introgression between wild and cultivated potato species. In Branch I, most accessions originated from Peru and belonged to clade 4, except for PG1013 (*S. pinnatisectum*), which originated in Mexico and belonged to clade 1 [3]. Branch II consisted entirely of accessions from South America, including Peru, Argentina, and Bolivia, all within clade 4. Branch III exhibited the most diverse origins, encompassing South American countries such as Argentina, Bolivia, Brazil, Ecuador, Peru, and Uruguay, as well as North American regions, including Guatemala, Honduras, Mexico, and the USA. Accessions in this branch were distributed across clades 1, 3, and 4. Interestingly, the two *Etuberosum* species (*S. etuberosum* and *S. palustre*) were also grouped within Branch III. Collectively, these results highlight a strong association between corolla color regulatory genes and the evolutionary history of wild and cultivated potato species.

### 2.5. Transcriptional Factor(s) Regulate the Accumulation of Anthocyanins by Modulating the Expression of Structural Genes in the Anthocyanin Biosynthetic Pathway

**Figure 5:**
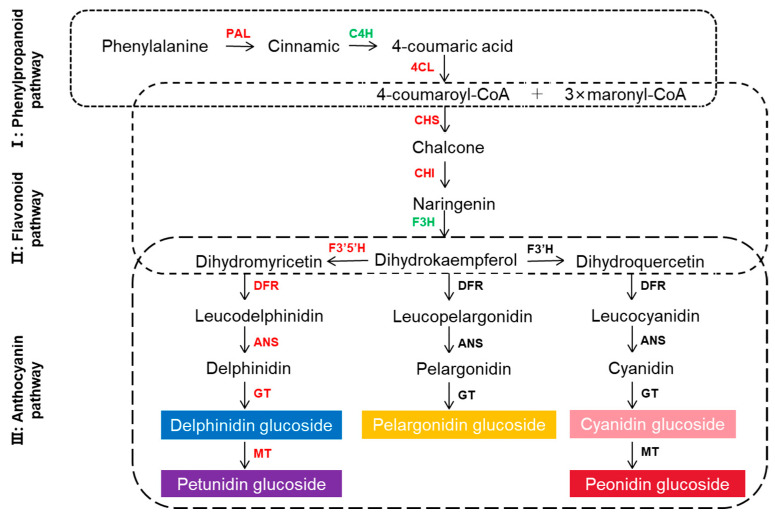
Expression patterns of structural genes in the anthocyanin biosynthesis pathway. Significant upregulated structural genes in the purple corolla pool compared to the yellow corolla pool are indicated in red. Cinnamate 4-hydroxylase (C4H) and flavonoid 3-hydroxylase (F3H), which were not differentially expressed but showed upregulation in purple corollas relative to yellow corollas, are marked in green. The following genes are annotated: PAL: Phenylalanine ammonia-lyase; C4H: Cinnamate 4-hydroxylase; 4CL: 4-coumarate-CoA ligase; CHS: Chalcone synthase; CHI: Chalcone isomerase; F3H: Flavonoid 3-hydroxylase; F3′H: Flavonoid-3′-hydroxylase; F3′5′H: flavonoid-3′5′-hydroxylase; DFR: Dihydroflavonol-4-reductase; ANS: Anthocyanidin synthase; GT: Glucosyltransferase; MT: Methyltransferase.

BSR-Seq analysis revealed that differentially expressed genes (DEGs) are significantly enriched in 20 pathways, including flavonoid biosynthesis (ko00941) and phenylpropanoid biosynthesis (ko00940). To elucidate the mechanism underlying the color formation of PH1-2 corollas, we examined the expression patterns of structural genes involved in the anthocyanin biosynthesis pathway (Figure 5). Notably, key genes such as *DM8C10G05870* (PAL) and *DM8C08G22170* (4CL), which are integral to the phenylpropanoid pathway, along with *DM8C05G00010* (CHS), *DM8C09G25980* (CHS), *DM8C02G23960* (CHI), *DM8C11G21030* (F3′5′H), *DM8C02G24950* (DFR), *DM8C08G26790* (ANS), *DM8C01G44370* (GT), *DM8C01G38690* (MT), and *DM8C03G25760* (MT) associated with flavonoid and anthocyanin pathways, exhibited significant upregulation in purple corollas compared to yellow corollas. Although cinnamate-4-hydroxylase (C4H) genes—*DM8C06G32340*, *DM8C06G32330*—and flavonoid 3-hydroxylase (F3H) genes—*DM8C03G36120*, *DM8C07G19220*, *DM8C03G13590*, and *DM8C07G19190*—showed no significant differential expression between the contrasting corolla color pools, they were nonetheless upregulated in purple corollas relative to yellow. These findings substantiate the hypothesis that *ScFL1* regulates anthocyanidin accumulation by modulating the expression of structural genes within the anthocyanin biosynthetic pathway.

## 3. Discussion

In previous work, we localized the *Fab* locus to a 740 kb region at the distal end of chromosome 10 using an F_1_ population derived from the crossing of the purple-colored abaxial of corolla genotype PH1-2 with the uniformly yellow-colored corolla genotype PH1-14 [16]. In the current study, we refined this locus to a 380 kb region through recombinant analysis, encompassing 28 genes according to the DMv8.1 reference genome [17]. Among these genes, five encode MYB transcription factors. Targeted measurements of anthocyanins and carotenoids in the corollas of PH1-2 and PH1-14 revealed that the purple anthocyanins present in the corollas of PH1-2 are primarily composed of delphinidin and petunidin glucosides, whereas the yellow pigments in the corollas of PH1-14 predominantly consist of β-carotene (Figure 2). These findings align with previous research on plant pigments.

MYB transcription factors play a vital role in regulating the flavonoid biosynthesis pathway in plants, influencing the coloration of stems, leaves, floral organs, and fruits [20]. Among the five MYB genes located in the identified region, *StMYB130* (*AN2*), a homolog of *StFlAN2* known to regulate anthocyanin production in corollas and linked to the *F* locus, does not exhibit significant differential expression between the contrasting corolla color pools. This suggests that *StMYB130* is unlikely to be responsible for the color variation observed between PH1-2 and PH1-14. On the other hand, *StMYB120* (*StMYB113-like*) and *StMYB170* (*MYB1-like*) have been associated with anthocyanin synthesis in potato tubers and onions [21,22]. Additionally, *StMYB200*, also referred to as *StMYBA1* or *Stan3*, has been identified as a candidate gene for the *I* locus, which is essential for anthocyanin accumulation in tuber skin [23]. Although *StMYB120*, *StMYB170*, and *StMYB200* display significant differential expression, their higher expression levels in yellow corollas compared to purple corollas indicate that they do not serve as candidates for the *Fab* locus. In contrast, *StMYB180*, another *StMYB113-like* gene, shows significantly higher expression in purple corollas relative to yellow, suggesting that it could be a candidate gene for the *Fab* locus. Further analysis confirmed that *StMYB180* exhibited high expression levels exclusively in the leaves and corollas of PH1-2, specifically in purple-colored abaxial tissues. These findings suggest that *StMYB180* plays a role in regulating tissue-specific anthocyanin biosynthesis in these regions. Moreover, the *Fab* locus has been linked to the *Pf* locus, which is essential for anthocyanin biosynthesis in tuber flesh, and the *B* locus, responsible for anthocyanin production in seed spots, the nodal band, the floral abscission layer, and tuber eyebrows [8,9]. Therefore, we hypothesize that the MYB genes residing within the *F-Fab-I-Pf-B* loci may collaboratively regulate anthocyanin accumulation across various potato tissues, including the sprout and stem, calyx, fruit, flower, and tuber.

In this study, we identified *StMYB180* as a regulator of anthocyanin biosynthesis in the abaxial corolla tissues. Phylogenetic analysis of *StMYB180* orthologs in 44 accessions revealed that most Branch I and II accessions originated from Peru, Argentina, and Bolivia, aligning with clade 4. In contrast, Branch III exhibited a more diverse geographic distribution, spanning six South American and four North American countries, with accessions distributed among clades 1, 3, and 4. The results of our phylogenetic study were compared with those of Tang et al. [19], and found that they were generally consistent. This consistency may be attributed to the fact that corolla color is not an economic trait in potato and has not undergone artificial domestication. As a result, it can better reflect the natural evolutionary process. However, we also found some differences, which may be influenced by interspecific hybridization. These findings support the hypothesis that corolla color is closely linked to the evolutionary pathways of wild and cultivated potato species. In order to attain more reliable and credible phylogenetic results, more species and materials with a high diversity of *Petota* section should be used in the future.

The biosynthesis of anthocyanins is well-conserved across plant species [24] and consists of three distinct stages (Figure 5). (1) The phenylpropanoid pathway, which begins with phenylalanine catabolism and involves a chalcone intermediary. (2) The flavonoid pathway, initiated by chalcone synthase (CHS), which catalyzes the step from 4-coumaroyl-CoA and malonyl-CoA to naringenin chalcone, which is then isomerized by chalcone isomerase (CHI) to form naringenin. (3) The anthocyanin pathway, where flavanone 3-hydroxylase (F3H) catalyzes naringenin to dihydrokaempferol. This compound can then undergo further hydroxylation by flavonoid 3′-hydroxylase (F3′H) or flavonoid 3′5′-hydroxylase (F3′5′H) to produce dihydroquercetin or dihydromyricetin. Subsequently, these intermediates are converted into colorless leucoanthocyanidins under the function of dihydroflavonol 4-reductase (DFR) and eventually transformed into colored anthocyanidins by anthocyanidin synthase (ANS). Our study demonstrated that the purple anthocyanins found in the corollas of PH1-2 are primarily derived from delphinidin and petunidin glucosides. Transcriptome analysis indicated that the structural genes associated with the biosynthetic pathways of these glucosides were expressed in the purple corolla pools, with several key genes—such as *DM8C10G05870* (PAL), *DM8C02G23960* (CHI), and *DM8C08G26790* (ANS)—showing significantly higher expression levels in purple corollas than in yellow corollas.

Corolla color not only serves as a crucial morphological marker for studying the evolution of closely related species but also provides a valuable resource for investigating functional differentiation among orthologous genes. In this study, we identified the *StMYB180* as a potential candidate gene controlling the potato corolla color, and in the future, we will focus on the interactions between *StMYB180* and structural genes to comprehensively analyze its regulatory mechanism. In conclusion, our research offers new insights into the regulation of anthocyanin biosynthesis in potatoes and provides a foundation for understanding the evolutionary dynamics of orthologous genes within the section *Petota*.

## 4. Methods and Materials

### 4.1. Plant Materials

Diploid accession PH1-2 was initially classified as *S. cardiophyllum* in the database maintained by the Institute of Vegetables and Flowers, Chinese Academy of Agricultural Sciences (IVF, CAAS), China. Through phenotype identification and genomic comparison, we determined that this accession is actually diploid *S. commersonii*, consistent with earlier instances of misidentification between these closely related species [25,26]. To investigate the *Fab* locus, PH1-2, characterized by purple abaxial corolla coloration, was crossed with PH1-14 (*S. cardiophyllum*), which has uniformly yellow corollas, to create an F_1_ segregation population [16]. In this study, we produced a larger F_1_ population comprising 279 individuals for further fine mapping of this locus. All materials used for mapping, BSR-Seq, and qPCR analyses were cultivated in an experimental field located in Zhangjiakou (40.82° N, 114.88° E) during the 2023 and 2024 growing seasons. Following collection, all plant tissues were immediately frozen in liquid nitrogen and stored at −80 °C until further analysis.

### 4.2. Targeted Measurement of the Anthocyanidins and Carotenoids

The content of anthocyanidins was quantified using the pH differential spectrophotometry method established by Wrolstand et al. [27]. Approximately 1 g of tissue was homogenized to a fine powder in liquid nitrogen and subjected to extraction with 5 mL of extraction solution composed of 0.05% HCl in methanol, maintained at 4 °C for 12 h. Following this, the mixture was centrifuged at 10,000× *g* for 20 min. The resulting supernatant was collected into a clean tube, and the sediment was re-extracted using an additional 5 mL of extraction solution at 4 °C for 6 h. This extraction process was repeated once more. The combined supernatants were measured to determine the final volume. Subsequently, 1 mL of the supernatant was mixed with 4 mL of either buffer A (0.4 M KCl, adjusted to pH 1.0 with 2 N HCl) or buffer B (1.2 N citric acid, adjusted to pH 4.5 with 0.2 M NaH_2_PO_4_), and the absorbance was recorded at 510 nm and 700 nm. The total anthocyanin content (TA), expressed as mg of cyanidin-3-O-glucose equivalent per 100 g, was calculated according to Romero et al. [28] using the formula: TA = A × MW × 5 × 100 × V/e, where A is defined as [A510 nm (pH 1.0) − A700 nm (pH 1.0)] − [A510 nm (pH 4.5) − A700 nm (pH 4.5)]. A molar absorptivity (e) value of 26,900 and a molecular weight (MW) of 449.2 were utilized, as per Wrolstand et al. [27]. Three measurements were conducted for each biological replicate sample.

The carotenoid content of the corollas was assessed using acetone extraction as described by Yin et al. [29]. Approximately 1 g of tissue was homogenized into a fine powder with liquid nitrogen. Carotenoids were extracted using a solvent mixture of ethanol and acetone in a 1:1 volume ratio until complete extraction was achieved. The resulting extract was then homogenized, and the volume was adjusted to 25 mL with the ethanol–acetone mixture. Absorbance measurements and the maximum absorption peaks were recorded at wavelengths of 663 nm, 645 nm, and 470 nm using a spectrophotometer, with each measurement performed in triplicate. The carotenoid concentration (Car) was calculated using the formula: Car = 8.73 × OD_470_ + 2.11 × OD_663_ − 9.06 × OD_645._

### 4.3. Fine Mapping and Candidate Gene Prediction

We designed 14 Indel primers within the previously identified mapping region located on chromosome 10 (53.11–53.85 Mb) utilizing the primer design module of OcBSA [16]. The primers’ polymorphism detection efficiency was initially evaluated using two parent lines. Subsequently, a small population comprising 60 F_1_ individuals along with the two parental lines was employed to assess the linkage between the markers and phenotypic traits. The linked markers were then applied to identify recombinants in a larger population of 279 F_1_ individuals. Genomic DNA extraction, PCR conditions, and genotyping were conducted via polyacrylamide gel electrophoresis, in accordance with previously established protocols [30]. The annotation data from the potato reference genome DMv8.1 [17] were utilized to perform functional analyses of all genes within the mapped interval to aid in the identification of candidate genes.

### 4.4. BSR-Seq

For the BSR-Seq analysis, fresh corollas were collected from 30 F_1_ individuals with purple corollas and 30 F_1_ individuals with yellow corollas, and then promptly frozen in liquid nitrogen. An equal amount of corolla tissue was pooled from each color group to create two sequencing libraries. RNA was extracted using the RNeasy Plant Mini Kit (Qiagen, Düsseldorf, Germany, Cat.# 74904) and subsequently utilized to construct libraries, which were sequenced on an Illumina Novaseq 6000 platform (Berry Genomics Beijing Co., Ltd., Beijing, China). The raw sequencing reads were processed using fastp for quality filtering to yield clean data. High-quality clean reads were aligned to the gap-free reference genome DMv8.1 [17] using Hisat2 [31], while gene expression levels were quantified employing StringTie v1.3.6 [32]. Gene expression was represented using fragments per kilobase of transcript per million fragments mapped (FPKM). Differentially expressed genes (DEGs) were identified based on the thresholds of |log_2_(fold-change)| ≥ 1 and false discovery rate (FDR) < 0.01.

### 4.5. qPCR Analysis

Corollas from the parental lines and two contrasting pools constructed for BSR-Seq were collected for quantitative PCR (qPCR) analysis to assess the expression of *StMYB180.* To further investigate the expression of *StMYB180* across various tissues, samples were obtained from five tissues: tuber, root, stem, leaf, and corolla. First-strand cDNA synthesis was conducted using 1 μg of total RNA and the HiScript^®^ III 1st Strand cDNA Synthesis Kit (Vazyme, Nanjing, China). *StMYB180* expression levels were quantified via qRT-PCR using gene-specific primers that amplify a sequence outside the targeted region (*StMYB180*_qPCR_F1/R1, Table S3). The qRT-PCR reactions were performed in a 10 µL volume consisting of 1 µL of diluted cDNA, 5 µL of SYBR qPCR Master Mix, and 0.2 μL of each forward and reverse primer (10 μM). Amplifications were conducted on a LightCycler480 II thermocycler (Roche, Ludwigsburg, Germany) with each reaction executed in triplicate. The *ef1α* gene, amplified using *ef1α*_F1/R1 (Table S3), served as the reference gene for normalizing gene expression data [33]. The primer efficiencies for both target and reference genes were within the range of 100–110%, enabling analysis of gene expression data using the 2^−ΔΔCt^ method [34]. Three biological replicates were utilized for the expression analysis.

### 4.6. Phylogenetic Analysis

To construct the neighbor-joining tree for the 26 species (44 accessions), amino acid sequences of the longest transcripts were extracted from 23 wild accessions, 19 landraces, and 2 *Etuberosum* species [19]. Protein sequences were aligned using MAFFT (v7.471) [35], and a phylogenetic tree was constructed using tvBOT (version 2.6.1) [36].

## Figures and Tables

**Table 1:** The nine identified DEGs in the *Fab* locus.

Gene ID	Chromosome	Start	End	Function Description
DM8C10G21040	Chr10	53,326,451	53,326,975	Belongs to the GRAS family
DM8C10G21090	Chr10	53,356,054	53,358,556	Belongs to the universal ribosomal protein uL23 family
DM8C10G21100	Chr10	53,367,231	53,382,114	Phosphatidylinositol 4-phosphate 5-kinase
DM8C10G21110	Chr10	53,382,925	53,384,556	Rab subfamily of small GTPases
DM8C10G21120	Chr10	53,384,808	53,385,801	Myb-like DNA-binding domain
DM8C10G21140	Chr10	53,490,901	53,492,318	Heavy-metal-associated domain
DM8C10G21170	Chr10	53,531,110	53,531,478	Myb-like DNA-binding domain
DM8C10G21180	Chr10	53,554,576	53,554,949	Myb-like DNA-binding domain
DM8C10G21200	Chr10	53,585,984	53,587,164	Myb-like DNA-binding domain

## Data Availability

The original contributions presented in this study are included in the article/Supplementary Material. Further inquiries can be directed to the corresponding authors.

## Supplementary Materials

The following supporting information can be downloaded at: https://www.mdpi.com/article/10.3390/ijms26051966/s1.
