# Supplementary figures and images for: Fine Mapping and Candidate Genes Analysis for Regulatory Gene of Anthocyanin Synthesis in the Corolla, Shedding Light on Wild Potato Evolution
Source: Int J Mol Sci. 2025 Feb 25;26(5):1966. doi: 10.3390/ijms26051966 (PMC11899842; doi:10.3390/ijms26051966)

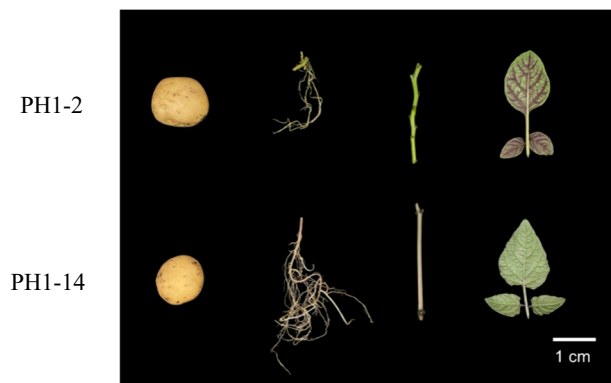

**Supplementary Figure S1. Organs of PH1-2 and PH1-14, including tuber, root, stem, and leaf.**

Supplement: Supplementary file 1 [file ijms-26-01966-s001.zip › Supplementary Figure S1.pdf]
